# Recurrent Uterine Smooth Muscle Tumor of Uncertain Malignant Potential in a Postmenopausal Woman After Total Abdominal Hysterectomy With Bilateral Salpingo‐Oophorectomy: A Case Report

**DOI:** 10.1155/crip/2301344

**Published:** 2026-07-22

**Authors:** Ania Mezouari, Yaritza Gonzalez Ruiz, Anthony Serici, Virginia Fernandez, Guido Santacana-Laffitte, Vangie Texidor

**Affiliations:** ^1^ Department of Clinical Sciences, University of Medicine and Health Sciences, Basseterre, Saint Kitts and Nevis, umhs-sk.org; ^2^ Department of Pathology, University of Miami Health System and Jackson Memorial Hospital, Miami, Florida, USA; ^3^ Department of Radiology, Hospital Español Auxilio Mutuo, San Juan, Puerto Rico, USA; ^4^ Department of Radiology, Virtual Radiologic (vRad), Eden Prairie, Minnesota, USA; ^5^ Department of Surgery, HCA Florida Mercy Hospital, Miami, Florida, USA

**Keywords:** case report, extrauterine recurrence, postmenopausal, recurrent STUMP, STUMP

## Abstract

**Background:**

Uterine smooth muscle tumors of uncertain malignant potential (STUMPs) are uncommon uterine neoplasms representing 1%–2% of uterine smooth muscle tumors. Recurrence after hysterectomy in postmenopausal patients has rarely been reported. Molecular alterations associated with recurrence remain poorly characterized. Methylthioadenosine phosphorylase (MTAP) deficiency has been described in sarcomas, but its role in STUMP remains poorly characterized.

**Case Presentation:**

A 61‐year‐old postmenopausal woman developed multifocal extrauterine recurrence affecting the rectus abdominis, vaginal cuff, peritoneum, small bowel, and abdominal wall 18 months after total abdominal hysterectomy with bilateral salpingo‐oophorectomy (TAH‐BSO) for presumed leiomyomas. The cumulative clinicopathologic findings were considered most consistent with recurrent STUMP, with one lesion exhibiting loss of MTAP expression. Retrospective review of the original hysterectomy specimen revealed previously unrecognized STUMP features, highlighting the diagnostic challenges at initial presentation.

**Conclusion:**

This case presents an unusual multifocal recurrence of STUMP following hysterectomy in a postmenopausal patient and reinforces the importance of long‐term surveillance, even after definitive surgical management. Focal MTAP loss was identified in one recurrent lesion and should be interpreted cautiously as a descriptive, hypothesis‐generating finding.

## 1. Introduction

Uterine smooth muscle neoplasms vary from benign leiomyomas to highly malignant leiomyosarcomas, with an intermediate (borderline) category known as STUMP when histological characteristics are unclear [1, 2]. The 2020 World Health Organization (WHO) states that STUMPs exhibit one or more atypical traits, including focal nuclear atypia or increased mitotic activity, along with other concerning features, without meeting the complete criteria for malignancy [1]. These tumors represent approximately 1%–2% of all uterine smooth muscle neoplasms in reported series [3, 4]. Reported recurrence rates range from 10% to 20%, and a subset of recurrences may meet criteria for leiomyosarcoma [5].

STUMP presents a management challenge, as hysterectomy is frequently regarded as the definitive treatment; however, recurrence may manifest as extrauterine disease, necessitating extended surveillance and ambiguity concerning optimal follow‐up intervals. Current management depends on total resection when possible and personalized imaging‐based monitoring, as there are no established pathological or molecular markers that consistently stratify recurrence risk [4, 5]. Recent studies have suggested that molecular and immunohistochemical alterations may serve as adjuncts to morphologic classification; however, data specific to STUMP remain limited [4, 6, 7].

In this case, MTAP loss was reported in one recurrent lesion, whereas MTAP status was not reported for the remaining recurrent specimens. MTAP, located at locus 9p21 and frequently co‐deleted with CDKN2A, is absent in numerous aggressive neoplasms and possesses therapeutic importance due to its susceptibility related to the PRMT5/MAT2A axis [8–10]. The clinical significance of MTAP/CDKN2A alterations in uterine smooth muscle tumors, including STUMP, remains poorly defined.

This report describes multifocal extrauterine recurrence of STUMP after TAH‐BSO in a postmenopausal patient and highlights the diagnostic challenges in evaluating initial hysterectomy specimens.

## 2. Case Presentation

A 61‐year‐old postmenopausal woman underwent a TAH‐BSO in May 2023 due to symptomatic leiomyomas, which had formed around a copper intrauterine device that had been retained for more than 30 years. Eighteen months later, she experienced worsening abdominal and pelvic pain. There was no history of gynecological cancer, hormone replacement therapy, or postmenopausal bleeding.

**Figure 1:**
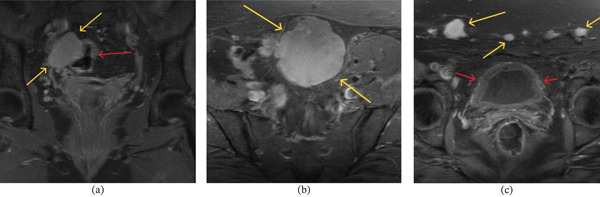
(a) Coronal T1‐weighted fat‐suppressed postcontrast image shows a 4.7 cm well‐defined, homogeneously enhancing mass (yellow arrows) abutting the sigmoid colon (red arrow), just cranial to the hysterectomy bed, without evidence of invasion into the adjacent structures. (b) Axial T1 fat‐suppressed postcontrast image shows a homogeneously enhancing mass (yellow arrows) centered along the midline with partial invasion of the left rectus abdominis muscle. The lesion measures approximately 7.4 × 7.0 cm. (c) Axial T1‐weighted fat‐suppressed postcontrast image shows multiple enhancing subcutaneous masses in the ventral pelvic wall (yellow arrows). The urinary bladder (red arrows) serves as a reference point.

Examination disclosed a large, palpable, solid, nontender mass in the left lower quadrant, adjacent to the prior Pfannenstiel incision. The pelvic MRI revealed numerous well‐defined, enhancing soft tissue masses. The scan demonstrated a 7.4 cm lesion invading the left rectus abdominis muscle, a 4.7 cm mass in the vaginal cuff, and multiple smaller nodules located subcutaneously and within the peritoneum (Figure 1A–C).

A core needle biopsy of the abdominal wall lesion revealed bland spindle cells arranged in intersecting fascicles, with an absence of atypia, mitotic activity, or necrosis. Given the limited sampling inherent to core needle biopsy, these findings did not exclude a more cellular or mitotically active component elsewhere in the lesion. Immunohistochemistry (IHC) showed positivity for desmin and smooth muscle actin (SMA), supporting smooth muscle differentiation. WT1 and estrogen receptor (ER) positivity were considered compatible with Müllerian‐type origin in the clinical context. The negative markers helped exclude selected nongynecologic mesenchymal differential diagnoses. Internal controls were adequate for all IHC stains. The Ki‐67 labeling index was 2%. Additional immunostains for keratin AE1/3, S100, CD34, SOX10, HMB45, and p40 were negative.

**Figure 2:**
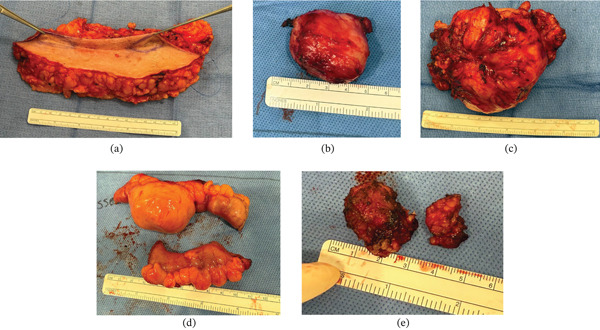
(a) Macroscopic view of the excised abdominal wall mass displaying multiple nodules, the largest measuring 3 cm. (b) Macroscopic appearance of the excised right lower abdominal mass, measuring 4.4 cm. (c) Macroscopic appearance of the excised lower mid‐abdominal wall mass, measuring 10 cm. (d) Macroscopic appearance of excised small bowel masses (three nodules, largest measuring 4.5 cm). (e) Macroscopic appearance of excised peritoneal masses.

Given the multifocal tumor burden, the patient underwent surgical resection of multiple grossly visible lesions for local disease control, including lesions from the rectus abdominis, vaginal cuff, peritoneum, small bowel, and subcutaneous tissue and abdominal wall (Figure 2A–E). During the operation, 18 cm of small intestine was resected with a primary anastomosis, and Phasix mesh was placed to augment the fascial closure.

Histological analysis of the excised specimens showed spindle cell tumors with moderate cytologic atypia, absent coagulative tumor cell necrosis, and focally increased mitotic activity of up to 20 mitoses per 10 high‐power fields (HPFs) as reported in the original pathology evaluation; field diameter and field area were not available for standardized conversion to square millimeters, identified in mitotic “hot spots” with interlesional variability.

IHC confirmed smooth muscle differentiation, demonstrating positivity for ER, SMA, and desmin, whereas p53 exhibited a wild‐type expression pattern, and expressions of Rb, PTEN, and ATRX were retained. MDM2 expression was not. The Ki‐67 index in the resected specimens was approximately 10%. Specimen C showed loss of MTAP expression as reported in the postoperative pathology report; the MTAP‐stained slide was not available for independent review, and internal control staining could not be directly assessed. MTAP status was not reported for the remaining recurrent specimens. All specimens had negative margins, except for Lesion A, which had a focally positive deep margin and showed venous and adjacent skeletal muscle invasion. The postoperative pathologic findings of the recurrent lesions are summarized in Table 1.

**Table 1:** Postoperative pathological characteristics of excised tumors.

Specimen (involvement)	Size (cm)	Mitotic rate (per 10 HPFs)^a^	Cytologic atypia/necrosis	Margins	Venous invasion	MTAP status	Ki‐67 (%)	Other molecular markers
A (dermis and subcutis)	Multiple: 3.0 (largest)	Up to 20	Mild–moderate/none	**Deep margin positive**	**Present**	Not reported	~10	N/D
B (intramuscular)	4.4	Up to 20	Mild–moderate/none	Negative (< 0.1 cm)	Absent	Not reported	~10	N/D
C (intramuscular)	10.0	Up to 20	Mild–moderate/none	Negative (< 0.1 cm)	Absent	**Lost**	~10	**PTEN, Rb, ATRX retained; MDM2 negative**
D (muscularis propria and subserosal fat)	Multiple: 4.5 (largest)	Up to 20	Mild–moderate/none	Negative	Absent	Not reported	~10	N/D
E (N/D)	N/D	Up to 20	Mild–moderate/none	Extends to the cauterized margin	Absent	Not reported	~10	N/D

*Note:* For Specimen E, the anatomic site and size were not documented in the available pathology report. Bold text indicates findings considered particularly relevant to recurrence.

Abbreviation: N/D, not documented.

^a^HPF field diameter and field area were not available in the original pathology documentation; mitotic activity is therefore reported as documented in the pathology report.

**Figure 3:**
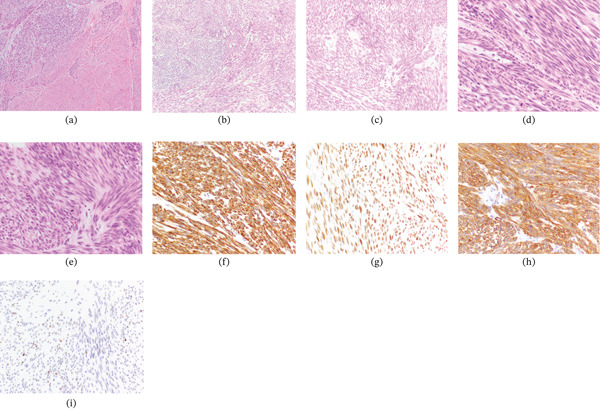
Comparative histologic and immunohistochemical features of the original hysterectomy specimen and recurrent lesions. (A–E) Comparative H&E findings: (a) H&E‐stained section from the original hysterectomy specimen showing smooth muscle tumor infiltration into the myometrium (20x). (b) H&E‐stained low‐power section of a recurrent lesion showing a hypercellular smooth muscle tumor (10x). (c) H&E‐stained medium‐power section of a recurrent lesion showing scattered mitotic activity (20x). (d) H&E‐stained high‐power section of a recurrent lesion showing scattered mitoses and mild cytologic atypia (40x). (e) H&E‐stained high‐power section from a different recurrent tumor focus showing similar mild atypia and mitotic activity (40x). (F–I) Immunohistochemical features of the recurrent lesions: (f) desmin immunostain showing positive tumor cell expression in a recurrent lesion (40x). (g) ER immunostain showing positive tumor cell expression in a recurrent lesion (40x). (h) SMA immunostain showing positive tumor cell expression in a recurrent lesion (40x). (i) p53 immunostain showing a wild‐type expression pattern in a recurrent lesion (40x).

Although the original hysterectomy specimen had initially been classified as benign, retrospective slide review demonstrated morphologic features overlapping with the recurrent lesions, including focal atypia, increased mitotic activity, absence of coagulative tumor cell necrosis, and infiltrative growth into the myometrium, supporting retrospective reclassification as STUMP. Representative comparative histologic images are shown in Figure 3.

The second surgery was performed in May 2025. Postoperatively, the patient was referred to gynecologic oncology for continued semiannual imaging surveillance. A CT scan performed 6 weeks after surgery showed no evidence of residual or recurrent disease. At the time this case report was prepared, no additional imaging or pathology documentation was available for complete clinicopathologic correlation. The key clinical events are summarized in Table 2.

**Table 2:** Timeline of key clinical events.

Time point	Clinical event
May 2023	TAH‐BSO was performed for presumed leiomyomas
18 months later	The patient developed worsening abdominal and pelvic pain; imaging revealed multifocal extrauterine lesions
May 2025	Second surgery: Lesions involving the rectus abdominis, vaginal cuff, peritoneum, small bowel, and abdominal wall were resected
6 weeks postoperatively	CT scan showed no evidence of residual or recurrent disease
At the time of manuscript preparation	No additional follow‐up imaging or pathology documentation was available for complete clinicopathologic correlation

## 3. Discussion

The differential diagnosis for multifocal extrauterine smooth muscle tumors includes parasitic leiomyoma, disseminated peritoneal leiomyomatosis (DPL), benign metastasizing leiomyoma (BML), and other forms of smooth muscle tumor dissemination [11, 12]. These conditions were carefully considered because the initial core needle biopsy showed bland spindle cells without atypia, mitotic activity, or necrosis, with a low Ki‐67 index (~2%), despite multifocal disease involving the peritoneum, abdominal wall, vaginal cuff, and small bowel. However, the excised lesions showed more concerning features, including moderate cytologic atypia, mitotic “hot spots,” venous invasion, infiltrative growth, and interlesional heterogeneity. Retrospective slide review of the original hysterectomy specimen also showed focal atypia, increased mitotic activity, and infiltrative growth into the myometrium, supporting retrospective reclassification as STUMP. Taken together, the clinicopathologic findings favored recurrent STUMP over a benign disseminated smooth muscle proliferation. Leiomyosarcoma was not favored because the lesions lacked coagulative tumor cell necrosis and showed no molecular or immunohistochemical profile supportive of high‐grade malignancy, with p53 wild‐type expression, retained Rb/PTEN/ATRX expression, and no MDM2 amplification [13]. The diagnostic framework for this classification is summarized in Table 3. The discrepancy between the biopsy and excision specimens was interpreted cautiously and likely reflects core biopsy sampling limitations and intratumoral heterogeneity.

**Table 3:** Diagnostic framework used for classification of the recurrent lesions.

1. SMA and desmin positivity supported smooth muscle differentiation; ER positivity was consistent with Müllerian‐type smooth muscle differentiation 2. Features of concern included mild to moderate cytologic atypia and focally increased mitotic activity (up to 20 mitoses per 10 HPFs) 3. Features not supportive of leiomyosarcoma were the absence of coagulative tumor cell necrosis, p53 wild‐type expression pattern, preserved Rb/PTEN/ATRX expression, and absence of MDM2 amplification 4. The combined morphologic and immunohistochemical features were more consistent with a diagnosis within the spectrum of STUMP rather than leiomyosarcoma

DPL was an important consideration because it can present as multifocal peritoneal or pelvic smooth muscle lesions and mimic malignancy or recurrence on imaging. Młodawski et al. described DPL following laparoscopic supracervical hysterectomy with uncontained mechanical morcellation and discussed dissemination of uterine/myometrial tissue fragments as a proposed mechanism, whereas metaplastic transformation of stem cells has also been suggested [14]. However, the present patient had no history of morcellation, making iatrogenic tissue dispersion less likely.

Lesion A had a positive deep margin; however, current literature has not shown a consistent association between margin status and STUMP recurrence, as recurrences have been reported even after complete excision with negative margins [5, 12]. In addition, the absence of exogenous hormonal stimulation makes hormonally driven recurrence less likely [15].

Focal loss of MTAP expression was identified in one recurrent lesion, whereas MTAP status was not reported for the remaining specimens. Because the MTAP‐stained slide was not available for independent review, this finding is presented cautiously as reported in the postoperative pathology report. Although MTAP loss may serve as a surrogate marker for CDKN2A deletion in some sarcoma contexts, including uterine leiomyosarcoma, its prognostic or therapeutic relevance in STUMP remains unestablished [11, 13]. Therefore, this finding should be interpreted cautiously as descriptive and hypothesis‐generating rather than risk‐defining or therapeutically actionable.

Compared with prior reports, this presentation was unusual. Most reported STUMP recurrences arise in premenopausal patients and generally manifest as localized pelvic or vaginal cuff lesions [4, 5]. In contrast, this postmenopausal patient developed multifocal disease involving the peritoneum, abdominal wall, and small intestine. Small bowel involvement was particularly distinctive because the lesion involved the muscularis propria and subserosal fat on pathologic examination, supporting true bowel wall involvement rather than gross adhesion alone [4, 5]. This pattern supports the notion that STUMP recurrence may extend beyond anticipated anatomical distributions, especially in postmenopausal cases.

The mechanism underlying multifocal recurrence in this case remains uncertain. Possible explanations include hematogenous dissemination, microscopic implantation, or growth of residual tumor foci; however, these mechanisms cannot be definitively established from the available clinical and pathologic data. Venous invasion raises the possibility of vascular spread, although this remains unproven [4, 5]. Therefore, the multifocal pattern is best interpreted cautiously as reflecting the unpredictable biology of STUMP rather than a proven route of dissemination. And although tumor cells exhibited ER positivity, ER expression indicates receptor presence rather than subsequent biological activity. In the absence of external hormonal stimulation, ER expression has not been reliably linked to recurrence or negative outcomes in STUMP [15]. Collectively, these findings suggest that recurrence in this case is most consistent with intrinsic tumor biology rather than modifiable surgical or hormonal factors.

In conclusion, this case demonstrates that STUMP can recur as multifocal extrauterine disease following TAH‐BSO in a postmenopausal patient, supporting the need for long‐term surveillance, even following definitive surgical treatment. MTAP loss was identified in one recurrent lesion and should be interpreted cautiously as a descriptive, hypothesis‐generating finding until its prognostic or therapeutic relevance in STUMP is established.

## Author Contributions

**Dr. Ania Mezouari** authored the manuscript, performed the literature review, facilitated communication with surgical and pathology teams, and submitted the manuscript. **Yaritza Gonzalez Ruiz** conducted patient interviews, obtained radiologic images, and assisted in the literature review. **Anthony Serici** performed a literature review, enhanced the differential diagnosis section, and contributed to manuscript editing and reference validation. **Dr. Virginia Fernandez** offered pathology expertise. **Dr. Guido Santacana-Laffitte** provided expert radiologic interpretation. **Dr. Vangie Texidor** performed the surgery, provided operative details, and oversaw manuscript preparation.

## Funding

No funding was received for this manuscript.

## Consent

Informed consent was collected from the patient for the publication of this case and its related images.

## Conflicts of Interest

The authors declare no conflicts of interest.

## Patient Perspective

Posthysterectomy, the patient experienced recurrent pelvic and abdominal pain, which was unexpected considering the anticipated definitive outcome of the initial procedure. Six weeks postsecond resection, she reported considerable symptomatic improvement and relief.

## Supporting information


**Supporting Information** Additional supporting information can be found online in the Supporting Information section. Supporting Information. File S1: CARE checklist for this case report.

## Data Availability

Data sharing is not applicable to this article as no datasets were generated or analyzed during the current study.
