# Supplementary material for: Recurrent Uterine Smooth Muscle Tumor of Uncertain Malignant Potential in a Postmenopausal Woman After Total Abdominal Hysterectomy With Bilateral Salpingo‐Oophorectomy: A Case Report
Source: Case Rep Pathol. 2026 Jul 22;2026:2301344. doi: 10.1155/crip/2301344 (PMC13392516; doi:10.1155/crip/2301344)
Supplement: Supplementary file 1 — Supporting Information Additional supporting information can be found online in the Supporting Information section. Supporting Information. File S1: CARE checklist for this case report. [file CRIP-2026-2301344-s001.docx]

| **CARE Section Completed** | **Checklist Item** | **Entry for This Case Report** | **Manuscript Location / Notes** |
| --- | --- | --- | --- |
| **Title** | The diagnosis or intervention of primary focus followed by the words “case report”. | Recurrent Uterine Smooth Muscle Tumor of Uncertain Malignant Potential (STUMP) in a Postmenopausal Woman After Total Abdominal Hysterectomy With Bilateral Salpingo-Oophorectomy: A Case Report | Title page |
| **Key Words** | 2 to 5 key words that identify diagnoses or interventions in this case report, including “case report". | STUMP; recurrent STUMP; postmenopausal; extrauterine recurrence; case report | Abstract page / Keywords |
| **Abstract** | Structured or unstructured abstract. | The abstract summarizes the background, case presentation, and conclusion. It describes an uncommon multifocal extrauterine recurrence of STUMP in a 61-year-old postmenopausal woman 18 months after TAH-BSO, with one recurrent lesion showing MTAP loss. | Abstract |
|  | The patient’s main concerns and important clinical findings. | The patient developed worsening abdominal and pelvic pain. Physical examination revealed a large, palpable, solid, non-tender mass in the left lower quadrant adjacent to the prior Pfannenstiel incision | Case Presentation |
|  | The primary diagnoses, interventions, and outcomes. | The primary diagnosis was recurrent STUMP. The patient underwent surgical resection of multiple lesions, including rectus abdominis, vaginal cuff, peritoneal, small bowel, subcutaneous, and abdominal wall lesions. A 6-week postoperative CT scan showed no evidence of residual or recurrent disease, and the patient reported symptomatic improvement. | Case Presentation / Follow-up |
|  | Conclusion: What are one or more take-away lessons from this case report? | STUMP can recur as multifocal extrauterine disease even after definitive hysterectomy in a postmenopausal patient. Long-term surveillance is important. Focal MTAP loss should be interpreted cautiously as a descriptive, hypothesis-generating finding. | Conclusion / Discussion |
| **Introduction** | Briefly summarizes why this case is unique and may include medical literature references. | The case highlights the diagnostic difficulty of STUMP at initial hysterectomy and demonstrates that recurrence may occur in unusual multifocal extrauterine sites after TAH-BSO. | Introduction |
| **Patient Information** | De-identified patient specific information. | A 61-year-old postmenopausal woman. | Case Presentation |
|  | Primary concerns and symptoms of the patient | Worsening abdominal and pelvic pain eighteen months after TAH-BSO. | Case Presentation |
|  | Medical, family, and psychosocial history including relevant genetic information. | The patient had no reported history of gynecologic cancer, hormone replacement therapy, or postmenopausal bleeding. Family and psychosocial history were not reported. Tumor-related molecular/IHC findings included p53 wild-type expression, retained Rb/PTEN/ATRX expression, no MDM2 amplification, and MTAP loss in one recurrent lesion. | Case Presentation / Pathology |
|  | Relevant past interventions and their outcomes. | The patient underwent TAH-BSO in May 2023 for presumed leiomyomas. The original hysterectomy specimen was initially classified as benign, but retrospective slide review later supported reclassification as STUMP. | Case Presentation |
| **Clinical Findings** | Describe significant physical examination and important clinical findings. | Physical examination showed a large, palpable, solid, non-tender mass in the left lower quadrant adjacent to the prior Pfannenstiel incision. MRI showed multiple enhancing soft tissue masses, including a 7.4 cm lesion involving the left rectus abdominis muscle, a 4.7 cm vaginal cuff mass, and additional subcutaneous and peritoneal nodules. | Case Presentation / Figure 1 |
| **Timeline** | Historical and current information from this episode of care organized as a timeline. | May 2023: TAH-BSO for presumed leiomyomas. Eighteen months later: worsening abdominal and pelvic pain with imaging showing multifocal extrauterine lesions. May 2025: surgical resection of lesions involving the rectus abdominis, vaginal cuff, peritoneum, small bowel, and abdominal wall. Six weeks postoperatively: CT showed no residual or recurrent disease. At manuscript preparation, no additional imaging or pathology documentation was available. | Table 2 |
| **Diagnostic Assessment** | Diagnostic methods. | Physical examination, pelvic MRI, core needle biopsy, histopathologic examination, immunohistochemistry, postoperative pathology evaluation, retrospective review of the original hysterectomy specimen, and postoperative CT imaging. Laboratory testing was not reported. | Case Presentation / Figures / Tables |
|  | Diagnostic challenges. | Diagnostic challenges included the initial benign classification of the hysterectomy specimen, bland findings on core needle biopsy, low Ki-67 on biopsy, inter-lesional heterogeneity, and difficulty distinguishing recurrent STUMP from parasitic leiomyoma, disseminated peritoneal leiomyomatosis, benign metastasizing leiomyoma, and leiomyosarcoma. | Discussion |
|  | Diagnosis, including other diagnoses considered. | Final diagnosis favored recurrent STUMP. Other diagnoses considered included parasitic leiomyoma, disseminated peritoneal leiomyomatosis, benign metastasizing leiomyoma, and leiomyosarcoma. Leiomyosarcoma was not favored because there was no coagulative tumor cell necrosis and no supportive high-grade molecular/IHC profile. | Discussion / Table 3 |
|  | Prognostic characteristics when applicable. | Prognostic features included multifocal extrauterine recurrence, venous invasion in lesion A, focal positive deep margin in lesion A, mitotic activity up to 20 mitoses per 10 HPF in hot spots, and MTAP loss in one recurrent lesion. The prognostic significance of MTAP loss in STUMP remains uncertain. | Table 1 / Discussion |
| **Therapeutic Intervention** | Types of therapeutic intervention. | Surgical intervention. The patient underwent resection of multiple grossly visible lesions for local disease control. Preventive/follow-up intervention included referral to gynecologic oncology for semiannual imaging surveillance. | Case Presentation |
|  | Administration of therapeutic intervention. | Surgical resection included lesions from the rectus abdominis, vaginal cuff, peritoneum, small bowel, subcutaneous tissue, and abdominal wall. Eighteen centimeters of small intestine were resected with primary anastomosis, and Phasix mesh was placed to augment fascial closure. No pharmacologic treatment dosage, strength, or duration was reported. | Case Presentation |
|  | Changes in therapeutic interventions with explanations. | Initial management was TAH-BSO for presumed leiomyomas. After multifocal recurrence was identified, management changed to surgical resection of recurrent lesions for local disease control, followed by planned semiannual imaging surveillance. | Case Presentation / Timeline |
| **Follow-up and Outcomes** | Clinician- and patient-assessed outcomes if available. | Clinician-assessed outcome: 6-week postoperative CT showed no residual or recurrent disease. Patient-assessed outcome: the patient reported considerable symptomatic improvement and relief six weeks after the second resection. | Case Presentation / Patient Perspective |
|  | Important follow-up diagnostic and other test results. | A six-week postoperative CT scan showed no evidence of residual or recurrent disease. No additional imaging or pathology documentation was available at the time the manuscript was prepared. | Case Presentation / Table 2 |
|  | Intervention adherence and tolerability. How was this assessed? | Adherence to follow-up surveillance was planned through gynecologic oncology. Tolerability was indirectly supported by the patient’s reported symptomatic improvement after surgery. Formal adherence or tolerability assessment was not reported. | Patient Perspective / Not formally assessed |
|  | Adverse and unanticipated events. | The unanticipated clinical event was multifocal recurrence after hysterectomy. No postoperative adverse events were reported. | Case Presentation / Discussion |
| **Discussion** | Strengths and limitations in our approach to this case. | Strengths include detailed clinicopathologic correlation, histologic and immunohistochemical evaluation of recurrent lesions, retrospective review of the original hysterectomy specimen, and consideration of important differential diagnoses. Limitations include limited original hysterectomy documentation, unavailable MTAP-stained slide for independent review, MTAP testing reported in only one lesion, and lack of longer-term follow-up imaging at manuscript preparation. | Discussion |
|  | Discussion of the relevant medical literature. | The discussion compares this case with reported STUMP recurrences, which more often occur in premenopausal patients and localized pelvic or vaginal cuff sites. It also discusses parasitic leiomyoma, disseminated peritoneal leiomyomatosis, benign metastasizing leiomyoma, leiomyosarcoma, and the uncertain role of MTAP/CDKN2A alterations in STUMP. | Discussion / References |
|  | The rationale for your conclusions. | Recurrent STUMP was favored because excised lesions showed mild to moderate atypia, mitotic hot spots, venous invasion, infiltrative growth, inter-lesional heterogeneity, and overlap with the retrospectively reviewed hysterectomy specimen. Leiomyosarcoma was not favored because coagulative tumor cell necrosis was absent and p53, Rb/PTEN/ATRX, and MDM2 findings did not support a high-grade profile. | Discussion / Table 3 |
|  | The primary take-away lessons from this case report in a one-paragraph conclusion. | STUMP may recur as multifocal extrauterine disease even after TAH-BSO in a postmenopausal patient. Long-term surveillance remains important because recurrence can occur outside expected pelvic sites. Focal MTAP loss should be reported cautiously and should not be overinterpreted as prognostic or therapeutically actionable without further evidence. | Conclusion |
| **Patient Perspective** | The patient should share their perspective on the treatment(s) they received. | Post-hysterectomy, the patient experienced recurrent pelvic and abdominal pain, which was unexpected given the anticipated definitive outcome of the initial surgery. Six weeks after the second resection, she reported considerable symptomatic improvement and relief. | Patient Perspective |
| **Informed Consent** | The patient should give informed consent. Provide if requested. | Informed consent was obtained from the patient for publication of the case report and related images. | Patient Consent section |
